# Development of a Computerized Adaptive Test for Separation Anxiety Disorder Among Adolescents

**DOI:** 10.3389/fpsyg.2020.01077

**Published:** 2020-06-18

**Authors:** Yiyuan Hu, Yan Cai, Dongbo Tu, Yingying Guo, Siyang Liu

**Affiliations:** School of Psychology, Jiangxi Normal University, Nanchang, China

**Keywords:** separation anxiety disorder, adolescent, computerized adaptive testing, item response theory, *DSM-5*

## Abstract

**Background:**

Separation anxiety disorder (SAD) is one of the most common mental disorders among children and adolescents, and it may seriously affect their growth, daily life, and learning. Self-report scales have been used for diagnosis, which require lengthy testing and personnel.

**Methods:**

A total of 1,241 adolescents were recruited from 16 junior- and senior-high schools in China. The initial item bank was selected from classical SAD scales according to the *DSM-5*. First, the optimal model was selected using item response theory (IRT) according to data fit. Then, per the IRT analysis, items that did not meet the psychometric requirements were deleted (e.g., discriminating values < 0.2). Consequently, a computerized adaptive test (CAT) for SAD was formed (CAT-SAD).

**Results:**

An average of 17 items per participant was required to achieve and maintain a 0.3 standard error of measurement in the SAD severity estimate. The estimated correlation of the CAT-SAD with the total 68-item test score was 0.955. CAT-SAD scores were strongly related to the probability of a SAD diagnosis with the Separation Anxiety Assessment Scale—Child and Adolescent Version. Therefore, SAD could be accurately predicted by the CAT-SAD.

**Conclusions:**

Exploratory factor analyses revealed that SAD was unidimensional. The CAT-SAD, which has good reliability and validity and high sensitivity and specificity, provides an efficient test for adolescents with SAD as compared to standard paper-and-pencil tests. It can be used to diagnose varying degrees of SAD quickly and reliably and ease the burden on adolescents. Potential applications for inexpensive, efficient, and accurate screening of SAD are discussed.

## Introduction

Separation anxiety disorder (SAD) is one of the most common mental disorders among children and adolescents—and its frequently reported symptoms are separation-related distress, avoidance of being alone/without an adult, and distress when sleeping away from caregivers/home (Allen et al., 2010)—as well as among some parents and patients undergoing psychotherapy. Currently, SAD begins (on average) at age 8 years, and it may persist into mid-childhood or adolescence (Last et al., 1992; Costello et al., 2003). SAD brings difficulties for both children and caregivers including undue worry, sleep problems, stress in social and academic environments, and a variety of physical symptoms that lower quality of life (Brand et al., 2011). Symptoms typically persist for more than 4 weeks, significantly interfering with children’s daily learning, which hinders their growth and development such as in interpersonal communication and learning efficiency (Eisen and Schaefer, 2007; Chessa et al., 2012).

Recently, some studies (e.g., Kossowsky et al., 2012) tracked the anxiety disorders of children and adolescents and showed that SAD was persistent and patients deteriorated steadily. Moreover, Lipsitz et al. (1994) suggested that early separation anxiety may constitute a non-specific vulnerability to a wide range of anxiety disorders in adulthood, including panic disorder. Some separation anxiety is a normal part of development in children aged 1–3 years. The lifetime prevalence is between 4 and 7.6% (Kessler et al., 2005; Shear et al., 2006; Merikangas et al., 2010; Milrod et al., 2014), and Manicavasaga et al. (1997) suggest that it may be possible to identify adults whose SAD mirrors the constellation of symptoms observed in childhood, even though some of the specific features are modified by maturation. Therefore, the early detection and intervention treatment of separation anxiety among children and adolescents are vital.

The definition of SAD has undergone significant changes in the *Diagnostic and Statistical Manual of Mental Disorders, Fifth Edition* (*DSM-5*)—the most consequential being the lifting of the age restriction (i.e., 18 years old) to assign a diagnosis. Why do clinicians traditionally redefine applicable ages? Because the construct of SAD has long been central to developmental theories that exert a strong influence in guiding clinical practice. In psychoanalytic and attachment theories, SAD is regarded as representative of neurophysiological, psychological, and behavioral responses designed to protect children from danger by ensuring close relationships with adult caregivers, typically mothers (Battaglia et al., 2009). Within the development framework of attachment theory, heightened expressions of SAD are regarded as indicating disturbances in children’s working models or internal representations of attachment figures, which are shaped by past and ongoing bonding experiences with primary caretakers (Bowlby, 1960).

According to the *DSM-5* definition, separation anxiety refers to individuals’ separation anxiety concerning their family and the related developmental problems. Significant symptoms such as physical symptoms (vomiting, stomachaches, etc.), emotional symptoms (anxiety and fear), and social functioning problems (declined learning efficiency) present themselves when adolescents are separated from their caregivers. A description of SAD symptoms in the *DSM-5* is shown in Table 2. If individuals meet any three symptoms of SAD, and they persist for at least 4 weeks, they are considered to have SAD. SAD comprises a repertoire of neurophysiological, intrapsychic, and behavioral responses. Therefore, experts hold different ideas about the dimensions of SAD; for example, one study suggested that separation anxiety was a multidimensional trait and that it should be divided into six dimensions (Hahn et al., 2003). However, In-Albon et al. (2013) suggested that a two-factor structure provided an adequate fit for the Separation Anxiety Avoidance Inventory—Child Version (SAAI-C). While an exploratory factor analysis (EFA) of the structure of children’s separation anxiety revealed a two-factor structure, a confirmatory factor analysis showed that the correlation between the two factors was 0.62 in a school-aged sample [standard error (*SE*) = 0.05, *p* = 0.01; In-Albon et al., 2013]. In other words, these dimensions measure different domains of SAD, and there is a significant correlation between them; i.e., they measure different domains of the same trait.

This study considers the arguments in favor of and against this definition change in the hope of stimulating debate and research aimed at achieving a consensus. We aimed to show that separation anxiety is unidimensional and provide a new perspective to the cross-cultural study of SAD measurements by using a Chinese sample. In fact, the scales that measure separation anxiety in previous studies have been developed according to Classical Test Theory. The purpose of the norm-referenced test is to distinguish the degree of separation of anxiety by maximizing the total score of the scale. At this point, how much more appropriate is the difficulty of each item on the test, and is the difficulty distribution of the item wider or narrower? A computer adaptive test (CAT) based on item response theory (IRT) may solve this problem. Furthermore, this study developed an assessment instrument of SAD based on CAT (SAD-CAT) in hopes of providing an effective instrument to measure SAD. CAT is more than just effective, due to cost and time effectiveness, less need for personnel to administer the test, and accurate and efficient diagnosis.

Computer adaptive test is an effective and fast measurement to evaluate participants’ individual latent traits (θ). CAT starts with randomly selecting one item from the test database and then selects the next item with lower or higher difficulty/threshold according to the previous responses. The process will continue when the uncertainty of the estimation capability does not reach the set value, or it will stop when the number of items reaches the predefined threshold. The paradigm shift is to manage items of different lengths to provide limited information to participants, depending on their specific level of the latent trait. Concurrently, CAT allows researchers to adaptively select a small set of items from a multi-item test based on participants’ prior latent trait estimation. Although only a small number of items are administered during this process, the information is comparable to several items. Therefore, compared with traditional tests of fixed length and topic, CAT has many remarkable advantages: (a) the length and test items differ among individuals; (b) it can effectively solve problems including long testing times and ineffective information for participants; and (c) it can present scores immediately after the test and has several practical implications, including the American Graduate School Humanities Test, the American Graduate School Admission Test, the American Nurses’ License Test, the American Military Occupational Direction Test, and so on.

Adolescents usually complete self-reports with the help of computer technology; therefore, a computerized adaptive application is advantageous for use with teenagers. In medical diagnoses, mental disorders usually rely on patients’ self-reports (or report to the diagnostician) to assess disorder presence and severity. Therefore, it is important to help patients complete self-reports effectively and accurately.

Further, CAT and IRT have been widely used in education measurements and competency assessment; however, their use in the field of personality and mental health needs to be expanded. To the best of our knowledge, using CAT and IRT to effectively assess SAD has not yet been formally discussed in the literature. We wanted to use CAT to achieve the goal of developing shorter and more effective tools to measure SAD and analyze the characteristics of teenagers. Specifically, we aimed to develop a new tool, an alternative to traditional paper-and-pencil (P&P) testing, that measures SAD with CAT and to examine its accuracy, reliability, and effectiveness.

## Method

### Sample

A total of 1,241 Chinese adolescents were recruited from 16 junior- and senior-high schools across nine cities in China. All adolescents and their guardians provided informed consent to participate, and their privacy was protected. Any participant with language issues was assisted, and participants completed the tests anonymously. The survey consisted of basic demographic questions, SAD measurement items, and exclusion criteria (see Table 1). To screen out individuals who randomly responded, four lie-detection items were embedded in the survey. For example, for an original item of the child version of the Revised Child Anxiety and Depression Scale (RCADS-C) such as “I am afraid of being alone at home,” its corresponding lie-detection item was “I am not afraid of being alone at home.” Participants who responded to any one of the four paired items using the same answer were eliminated from analyses.

**TABLE 1:** The diagnostic criteria of separation anxiety disorder (SAD) in *DSM-5* and the initial item bank structure.

	Number of items
(1) Developmentally inappropriate and excessive fear or anxiety concerning separation from those to whom the individuals is attached, as evidenced by at least three of the following:	
(a) Recurrent excessive distress when anticipating or experiencing separation from home or from major attachment figures.	11
(b) Persistent and excessive worry about losing major attachment figures or about possible harm to them, such as illness, injury, disasters, or death.	12
(c) Persistent and excessive worry about experiencing an untoward event (e.g., getting lost, being kidnapped, having an accident, becoming ill) that causes separation from a major attachment figure.	10
(d) Persistent reluctance or refusal to go out, away from home, to school, to work, or elsewhere because of fear of separation.	11
(e) Persistent and excessive fear of or reluctance about being alone or without major attachment figures at home or in other settings.	14
(f) Persistent reluctance or refusal to sleep away from home or to go to sleep without being near a major attachment figure.	12
(g) Repeated nightmares involving the theme of separation.	12
(h) Repeated complaints of physical symptoms (e.g., headaches, stomachaches, nausea, vomiting) when separation from major attachment figures occurs or is anticipated.	11
(2) The fear, anxiety, or avoidance is persistent, lasting at least 4 weeks in children and adolescents and typically 6 months or more in adults.	
(3) The disturbance causes clinically significant distress or impairment in social, academic, occupational, or other important areas of functioning.	
(4) The disturbance is not better explained by another mental disorder, such as refusing to leave home because of excessive resistance to change in autism spectrum disorder; delusions or hallucinations concerning separation in psychotic disorders; refusal to go outside without a trusted companion in agoraphobia; worries about ill health or other harm befalling significant others in generalized anxiety disorder; or concerns about having an illness in illness anxiety disorder.	

Next, 1,161 respondents completed the P&P tests. Of those, 56 (5.60%) participants were eliminated owing to lie-detection items, and 15 (1.4%) participants were excluded owing to meeting any of the pre-established exclusion criteria: (1) adolescents had inappropriate fear or anxiety that persisted for at least 4 weeks; (2) clinically significant distress or impaired social, learning, work, or other important functions caused this inappropriate fear or anxiety; and (3) the inappropriate fear or anxiety was better explained by other mental disorders, like infantile autism, psychotic disorder, agoraphobia disorder, or generalized anxiety disorder. In addition, there were 76 (5.6%) partial completers—most of the missing values concerned gender, age, and region. The MissMech R package (Jamshidian et al., 2014) was employed to test the assumption that data were missing completely at random (Rubin, 1976).

After eliminating missing values using the listwise deletion method, the final sample comprised 1,014 (effective response rate = 81.71%) participants. Participants’ ages ranged from 12 to 18 years (mean age = 15.42 ± 1.57 years). All participants were of Chinese ethnicity, and 55.82% (*n* = 566) were male. Moreover, 21.40% (*n* = 217) of the sample were from urban areas. Participants’ demographics are shown in Table 2.

**TABLE 2:** Demographic characteristics (*N* = 1,014).

Variables	Category	Frequency	Percent (%)
Gender	Male	556	54.84
	Female	458	45.16
Age	Under 16 years	610	60.16
	16 and above	405	39.94
Region	Rural	797	78.56
	Urban	217	21.4

### Measures

Initially, we reviewed the contents of six questionnaires that are commonly used to measure SAD to develop an item bank: the SAAI-C, the Multidimensional Anxiety Scale for Children, the Separation Anxiety Assessment Scale—Child and Adolescent Version (SAAS-C), the Separation Anxiety Symptom Inventory (SASI), the Screen for Child Anxiety Related Disorders (SCARED), and the Spence Children’s Anxiety Scale—Child and Adolescent Version (SCAS-C).

#### Separation Anxiety Avoidance Inventory—Child Version

The SAAI-C (Schneider and In-Albon, 2005) is a 12-item self-report scale that is rated on a five-point scale ranging from 0 (*never*) to 4 (*always*). According to In-Albon et al. (2013), the internal consistency coefficients ranged from 0.81 to 0.84, and the test–retest reliability was 0.80 (*p* < 0.01) in a school-aged sample. Among a sample of 49 participants with SAD, the SAAI-C total score correlated significantly with the separation anxiety subscale of the SCAS (*r* = 0.49). In this study, Cronbach’s α was 0.86.

#### Separation Anxiety Assessment Scale—Child and Adolescent Version

The SAAS-C (Hahn et al., 2003), which is suitable for children aged 6–18 years, is a 34-item self-report scale. All items have a four-point rating scale ranging from 1 (*never*) to 4 (*all the time*). The SAAS-C has six subscales including fear of being alone (five items), fear of abandonment (five items), fear of physical illness (five items), being worried about calamitous events (five items), frequency of calamitous events (five items), and a safety signals index (nine items). The SAAS-C possesses good internal consistency: αs = 0.91 and 0.85 in Hahn et al. (2003), and in this study.

#### Separation Anxiety Symptom Inventory

The SASI (Silove et al., 1993) is a 22-item self-report scale, and all items are rated on a four-point scale: *always*, *often*, *occasionally*, and *never*. In Silove et al. (1993), the SASI construct validity with symptoms of SAD was 0.79 (*p* < 0.00l). In this study, the Cronbach’s α was 0.81.

#### Screen for Child Anxiety Related Disorders

The SCARED (Birmaher et al., 1999) is a 37-item self-report scale that measures anxiety disorders among children and adolescents aged 9–18 years. Each item is rated on a three-point scale ranging from 0 (*not true*) to 2 (*certainly true*). In Birmaher et al. (1999), the Cronbach’s α for the SCARED total score was 0.89, and its subscale αs ranged from 0.43 to 0.77. In this study, the SAD subscale Cronbach’s α was 0.73.

#### Spence Children’s Anxiety Scale—Child and Adolescent Version

The SCAS-C (Spence, 1998) is a 44-item self-report scale that was designed to assess children’s anxiety symptoms. Items are rated on a four-point scale ranging from *never* to *always*. There are six subscales reflecting six symptoms: social phobia (six items), panic disorder and agoraphobia (nine items), generalized anxiety disorder (six items), obsessive–compulsive disorder (six items), SAD (six items), and fear of physical injury (five items). The total score was summed to reflect overall anxiety symptoms. The SCAS possessed good internal consistency (total scale > 0.90; subscales = 0.60–0.90; Spence et al., 2003; Essau et al., 2011; Zhao et al., 2012). In this study, Cronbach’s α was 0.75.

#### RCADS

The RCADS (Chorpita et al., 2000) is a 47-item child self-report scale to assess anxiety and depression disorder symptoms. It is rated on a four-point scale (0 = *never* to 3 = *always*). In addition to a depression scale (10 items), the RCADS has five anxiety scales: separation anxiety (7 items), generalized anxiety (6 items), panic disorder (9 items), social phobia (9 items), and obsessive–compulsive (6 items). Cronbach’s α for the total RCADS-C total was 0.92, and Cronbach’s α for its subscales are as follows: 0.81 for separation anxiety, 0.82 for generalized anxiety, 0.89 for social phobia, 0.76 for panic disorder, 0.68 for obsessive–compulsive, 0.71 for depression, and 0.91 for total anxiety (Chorpita et al., 2000). In this study, Cronbach’s α was 0.86.

### Procedure

First, according to the symptom criterion of SAD as defined in the *DSM-5*, experts from Wuhan Mental Health Center judged which symptoms were measured by each item of the SAD scales, and items fitting at least one symptom criterion were considered for selection. Moreover, to ensure there were enough items measuring each symptom of SAD, according to content balance guidelines, experts selected items from these scales to form the initial item bank of the CAT-SAD. Second, participants completed the initial item bank via P&P testing, and their response data were used for later IRT analyses, construction of the final item bank, and CAT simulation research.

### Item Bank

We intended to keep the original scoring of all items to verify the effectiveness of each scale in a cross-culture setting. Ninety-three items of the above six measures met the criteria and comprised our initial CAT-SAD item bank. As shown in Table 1, each symptom was measured by at least 10 items, which indicated that there were sufficient items to cover all symptoms of SAD as defined in the *DSM-5*. Moreover, a series of analyses under the framework of IRT were performed to choose the acceptable items from the initial item bank, which embraced the unidimensionality test, item fit test, and differential item function detection.

#### Unidimensionality

Unidimensionality of the 93-item P&P version of the SAD from the above six measures was first demonstrated using an EFA. The ratio of the first eigenvalue to the second eigenvalue was greater than 3 in EFA indicating unidimensionality (Lord, 1980; Hattie, 1984), and the percentage of variance explained by the first factors exceeded 20% (Reckase, 1979). According to Nunnally (1978), who observed that factor loadings smaller than 0.30 should not be taken seriously and that ones smaller than 0.30 could easily be over-interpreted, we first eliminated items whose factor loadings on the first factor were below 0.30 to confirm acceptable unidimensionality of the dataset; then, the EFA was conducted again to test unidimensionality.

#### IRT Model Selection

We considered IRT models with polytomous items including the graded response model (GRM; Samejima, 1969), the nominal response model (NRM; Bock, 1972), and the generalized partial credit model (GPCM; Muraki, 1992). Akaike’s information criterion (AIC; Akaike, 1974) and the Bayesian information criterion (BIC; Schwarz, 1978) of the three models were employed to compare model fit. The smaller the value of the AIC or BIC, the better the model fit; thus, the IRT model with the smallest AIC and BIC value was chosen for the IRT analysis in this study.

#### Item Calibration

##### Item fit

Evaluating model fit generally requires an evaluation of both test and item fit. Test fit was evaluated for whether the selected model was consistent with the actual data at the test level; item fit was evaluated as whether the model was consistent with the actual data at the item level, which can be used to screen items in the test. Item fit was evaluated as an absolute fit test, and this kind of method calculates some statistics between the model to be selected and the actual data. The *S-X^2^* index (Orlando and Thissen, 2000, 2003) tested item-level fit. Items with a *p-*value of *S-X^2^* less than 0.05 were considered to have poor item fit and were deleted. The R package MIRT (version 1.29; Chalmers, 2012) was utilized to evaluate item fit.

##### Discrimination parameter

According to the IRT, the item discrimination parameter defined the degree to which an item distinguishes between individuals with similar scores. An item with a high discrimination parameter *t* is high quality and could more accurately estimate the potential characteristics of the participants in the test. In addition, item discrimination had an important impact on item information, which was used to decide which item was selected in the CAT environment; therefore, items with low discrimination (i.e., less than 0.8) were excluded from the initial item bank (Tan et al., 2018).

##### Differential item functioning

Measurement bias is an important indicator of the validity of a questionnaire survey, and qualified items had no measurement bias for different groups (region, gender, age, health condition). This study used a differential item functioning (DIF) analysis to evaluate the systematic error caused by group bias (Zumbo, 1999). We used ordinal logit regression analysis (Crane et al., 2006) under the optimal model through *R* package Lordif (version 0.3-3; Choi, 2015) based on test-level model fitting checks. Items with changes in McFadden’s pseudo *R*^2^ < 0.2 were deemed as DIF (Flens et al., 2017) and were deleted from the initial item bank. DIF was independently evaluated by region (rural, urban), gender (male, female), age (<16 years, ≥16 years), and health condition (SAD, normal) groups.

### CAT-SAD Simulation Study

We performed a simulation study with the 1,014 adolescents to investigate the properties of the developed item bank. We examined four properties: reliability, validity, sensitivity, and specificity.

We simulated a CAT in the item bank from the real responses obtained from adolescents’ P&P data. At the beginning of the CAT, we did not know prior information about the adolescents (Kreitzberg and Jones, 1980). The first item that the CAT simulation started on was randomly selected from the item bank (Magis and Barrada, 2017). Then, base item parameters and adolescents’ item responses estimated their SAD latent trait (θ) and measurement precision. Here, the expected *a posteriori* method (Bock and Mislevy, 1982) was used to update adolescents’ SAD latent trait (θ) based on their real P&P responses. The maximum Fisher information criterion (Baker, 1992) selection strategy was adopted to select the next question for adolescents in the simulation of CAT-SAD, and three different stopping rules were set: 0.3, 0.4, and 0.5, respectively. When measurement accuracy or the pre-set test length (i.e., 20 items) was reached, the program would terminate (Magis and Raiche, 2012).

#### CAT-SAD Properties

To evaluate CAT-SAD properties, three statistic criteria were investigated to evaluate test estimation accuracy: the number of items used, SE, and marginal reliability (Smits et al., 2011). The number of items used was the number of items each adolescent answered when completing the test. The SE for trait level can be defined as the reciprocal of the square root of the value of the test information function at that trait level (Magis and Raiche, 2012); the formula is defined as follows:

$$\text{SE}\,\left(\theta \right)=\frac{1}{\sqrt{I}\,\left(\theta_{i}\right)},\mathrm{in}\,\mathrm{which}\,I\,\left(\theta_{i}\right)\,\mathrm{is}\,\mathrm{the}\,\mathrm{test}\,\mathrm{information}\,\mathrm{at}\,\theta_{i}$$

The corresponding reliability *r*_*xx*_(θ_*i*_) of each individual can be derived via the following formula (Samajima, 1994) when the mean and standard deviation (SD) of the score are fixed to 0 and 1, respectively:

$$r_{x\,x}\,\left(\theta_{i}\right)=1-\frac{1}{I\,\left(\theta_{i}\right)}$$

#### Validity

Criterion-related validity refers to the degree to which the measure is consistent with its measurement objectives. Taking the total SAAS-C score as the criterion, the correlation between separation anxiety level (θ), as estimated by the CAT-SAD, and the criterion data calculated was regarded as the criterion-related validity of the CAT-SAD. The high correlation indicated that the CAT-SAD had good criterion-related validity. We also investigated the content validity of the CAT-SAD by analyzing whether the items in the final item bank adequately measured all symptoms of SAD as defined in the *DSM-5*.

#### Sensitivity and Specificity

In medical diagnosis, sensitivity and specificity are usually used as an important reference index for the accuracy of delimitation scores. Sensitivity refers to the probability of a patient being diagnosed with a disease, and specificity refers to the probability that ordinary people will be diagnosed without the disease (Smits et al., 2011). Here, sensitivity and specificity were used to investigate the predictive utility of the CAT-SAD. In addition, the Youden index (*YI* = sensitivity + specificity – 1) was also used to assess the effect of the diagnosis by CAT-SAD, which reflected the difference between the rate of true positives and false positives. The larger the value of *YI*, the better the diagnostic capacity (Schisterman et al., 2005).

To calculate sensitivity and specificity, participants were classified as SAD samples and non-SAD samples by the SAAS-C. Specifically, 40 participants with total SAAS-C scores ≥ 75 were classified as the SAD sample, while the other 974 participants with SAAS-C scores < 75 were classified as the non-SAD sample (Eisen and Schaefer, 2007).

## Results

### Item Bank

#### Unidimensionality

Results of unidimensionality showed that the factor loadings on the first factor were less than 0.3. After excluding the 15 items, the EFA was conducted to analyze unidimensionality with the remaining items. The results indicated that the ratio between the first eigenvalue of 25.08 and the second eigenvalue of 5.59 was 4.49, and the first factor accounted for 25.08% (more than 20%). The above results indicated that the remaining 78 items met unidimensionality.

#### IRT Model Selection

The IRT model with the smallest value of AIC and BIC was finally chosen and applied (see Table 3). The AIC and BIC values in the GRM were the smallest compared with the GPCM and NRM, which showed that the GRM fit the data better than the others. Accordingly, the GRM was selected as the IRT analysis for the CAT-SAD.

**TABLE 3:** Fitting models.

Model	AIC	BIC
GRM	140,506	142,026.8
GPCM	141,084.2	142,605
NRM	140,832.7	143,106.5

*GRM, graded response model; GPCM, generalized partial credit model; NRM, nominal response model; AIC, Akaike’s information criterion; BIC, Bayesian information criterion.*

#### Item Fit and DIF

Results of the *S-X^2^* suggested that two items (*p*s < 0.05) were deleted from the item bank. Regarding DIF, there were no items in the regional, sex, age, and health condition groups (all items’ McFadden’s pseudo *R*^2^ were less than 0.2). In addition, the discrimination values of 15 items were less than 0.8; thus, they were deleted from the item bank (Tan et al., 2018).

The remaining 68 items in the item bank met unidimensionality, fit the GRM well, possessed high discrimination, and had no DIF. Table 4 shows the estimated item parameter values of GRM in the item bank. The discrimination parameters showed considerable variation and similar patterns for all scales, ranging from 0.83 (Item 2, “I feared that one of my parents might come to harm when I was away from home”) to 2.14 (Item 51, “I am afraid to be alone in the house”). The threshold parameters showed considerable variation for all scales; for example, all four Likert items ranged from −1.12 (Item 2, “I imagined that monsters or animals might attack me when I was alone at night”) to 6.82 (Item 13, “I am afraid my family might abandon me”). Therefore, the final item bank of the CAT-SAD included 68 items after 25 items were excluded for the abovementioned psychometric reasons.

**TABLE 4:** Location and discrimination parameter values and the descriptive statistics of the responses of each item for the item bank.

Item number	Item parameters					Descriptive statistics of the responses			
	*a*	b1	b2	b3	b4	Mean	*SD*	Skewness	Kurtosis
1	0.98	–0.32	2.04	3.04		0.79	0.87	1.06	0.58
2	0.83	–0.3	1.68	2.67		0.35	0.75	2.37	5.05
3	0.9	–0.82	1.01	2.12		1.11	1.06	0.57	–0.89
4	1.02	–0.16	1.79	2.54		0.8	0.95	1.09	0.25
5	1.23	0.42	1.64	2.33		0.66	0.96	1.34	0.64
6	0.88	–1.12	0.73	1.8		1.27	1.1	0.37	–1.17
7	0.92	1.75	3.52	4.38		0.31	0.61	2.32	5.9
8	1.36	0.93	2.48	3.61		0.36	0.65	1.96	3.74
9	1.27	0.83	2.48	3.59		0.4	0.69	1.84	3.21
10	1.08	0.6	2.58	3.5		0.49	0.75	1.65	2.45
11	1.17	–0.41	1.47	2.76		0.86	0.88	0.83	–0.03
12	1.05	–0.42	1.59	2.92		0.86	0.89	0.86	–0.01
13	1.85	0.34	1.46	2.1		0.61	0.88	1.42	1.15
14	1.4	–0.33	1.2	1.95		0.92	0.98	0.87	–0.26
15	0.88	0.18	2.13	3.18		0.71	0.92	1.21	0.55
16	1.32	–0.51	1.31	2.36		0.91	0.9	0.81	–0.05
17	1.04	0.93	2.75	3.96		0.41	0.71	1.85	3.14
18	1.61	0.51	1.53	2.15		0.58	0.91	1.52	1.26
19	1.64	–0.31	1.38	2.34		0.82	0.84	0.92	0.32
20	1.52	–0.4	1.42	2.53		0.82	0.83	0.87	0.3
21	1.02	0.6	2.55	3.6		0.51	0.77	1.59	2.11
22	1.61	–0.12	1.16	1.93		0.84	0.97	0.95	–0.13
23	1.56	0.67	2.13	2.99		0.42	0.71	1.79	3.03
24	1.82	0.14	1.75	2.62		0.59	0.75	1.26	1.37
25	1.63	0.25	1.8	2.86		0.56	0.75	1.31	1.34
26	1.5	–0.55	1.3	2.35		0.9	0.86	0.8	0.11
27	1.18	1.29	2.76	3.95		0.3	0.63	2.31	5.26
28	1.29	0.97	2.29	3.09		0.39	0.74	2.05	3.75
29	1.64	0.15	1.59	2.29		0.65	0.86	1.3	1.01
30	1.45	0.17	1.82	2.65		0.62	0.81	1.33	1.27
31	1.48	0.89	2.45	3.12		0.35	0.66	2.15	4.85
32	1.55	1	2.42	3.21		0.32	0.63	2.24	5.21
33	1.5	1.55	2.75	3.49		0.2	0.54	3.13	10.55
34	1.53	1.08	2.59	3.58		0.29	0.58	2.27	5.56
35	1.52	1.27	2.81	3.81		0.24	0.53	2.51	6.97
36	1.75	1.29	2.57	3.39		0.22	0.54	2.76	8.35
37	1.38	0.24	1.84	2.66		0.62	0.84	1.35	1.21
38	1.31	1.66	3.1	3.84		0.2	0.53	3.17	11.07
39	1.1	–0.01	3.01			0.56	0.6	0.56	–0.61
40	1.46	0.73	3.02			0.34	0.53	1.22	0.48
41	1.48	0.28	2.15			0.51	0.64	0.89	–0.28
42	1.7	–0.2	1.43			0.72	0.72	0.48	–0.95
43	1.1	–1	1.74			0.88	0.67	0.14	–0.77
44	1.53	0.14	1.84			0.58	0.68	0.76	–0.58
45	0.87	–0.49	2.32			0.74	0.7	0.4	–0.9
46	1.07	1.14	3.43			0.31	0.54	1.56	1.5
47	1.01	0.79	3.38			0.39	0.58	1.19	0.41
48	1	1.28	3.5			0.3	0.55	1.64	1.74
49	0.95	1.3	3.38			0.32	0.58	1.64	1.65
50	1.39	0.51	2.45			0.43	0.61	1.09	0.15
51	1.95	0.49	2			0.43	0.61	1.14	0.23
52	1.92	0.36	2.04			0.46	0.61	0.98	–0.07
53	1.27	0.41	3.01			0.44	0.57	0.86	–0.26
54	1.24	0.93	3.19			0.32	0.54	1.42	1.07
55	1.58	0.55	1.95			0.45	0.66	1.17	0.15
56	1.62	0.29	2.03			0.5	0.64	0.91	–0.26
57	1.82	0.05	1.65			0.6	0.68	0.7	–0.65
58	1.37	–0.6	1.8			0.78	0.66	0.26	–0.75
59	1.7	–0.11	1.52			0.68	0.71	0.55	–0.86
60	1.26	–0.6	0.71	2.12	3.02	1.13	1.09	0.79	–0.01
61	1.32	–0.67	0.36	1.32	2.24	1.38	1.29	0.58	–0.77
62	1.11	–0.11	1.06	2.27	3.34	0.96	1.13	1.03	0.2
63	0.89	0.03	1.6	3.22	4.32	0.83	1.03	1.24	0.98
64	1.07	0.18	1.3	2.38	3.04	0.87	1.17	1.3	0.78
65	0.97	–0.45	0.58	1.41	2.25	1.38	1.44	0.64	–0.98
66	1.55	0.14	0.92	1.74	2.29	0.92	1.22	1.19	0.35
67	1.38	–0.31	0.7	1.74	2.59	1.12	1.2	0.85	–0.23
68	1.38	–0.27	0.66	1.65	2.47	1.12	1.22	0.86	–0.29

**a* is the discrimination parameter; the *b*s are location parameters. Mean is the mean of all the participants’ response in each item, SD is the standard deviation of all the participants’ response in each items, skewness is the skewness of all the participants’ response in each item, and kurtosis is the kurtosis of all the participants’ response in each item.*

### CAT-SAD Simulation Study

#### Properties of the CAT-SAD

A description of the termination rules and the results are provided in Table 5. A CAT algorithm was run with no termination rules (“none” in Table 5) to generate scores based on administration of the full item bank for comparison. Table 5 reveals that the stop rule with the SE was less than 0.3 [i.e., SE (θ) < 0.3], an average of 17.04 items per participant was required with a marginal reliability of 0.89, and the correlation between the 17-item average CAT severity score and the total 68-item score was 0.953. In this study, seeking for a reliable and shorter measure, we specified that when the SE < 0.3, the CAT simulation terminated the latent trait estimate of an adolescent, and the marginal reliability was 0.89 (Green et al., 1984). Table 5 also indicated that, as the SE increased (i.e., less precise), the average amount of items decreased. For example, when SE increased from 0.3 to 0.4, the number of items, on average, decreased from 17.04 to 10.89, and the marginal reliability also decreased.

**TABLE 5:** Characteristics of the computerized adaptive test (CAT) under several stopping rules.

Stopping rule	Number of items used		Mean *SE* (θ)	Marginal reliability	Correlation^b^
	Mean	*SD*			
*None*	68	0	0.19	0.96^a^	1.000
*SE (θ) < 0.3*	17.04	2.43	0.31	0.89	0.953
*SE (θ) < 0.4*	10.89	3.96	0.40	0.84	0.924
*SE (θ) < 0.5*	7.456	3.61	0.48	0.77	0.892

*None = all of the item bank was used. ^*a*^Coefficient alpha for the full test was 0.960. ^*b*^Correlation between CAT_θ_ and complete test θ.*

The descriptive statistics of the responses to each item in the final item bank are presented in Table 4. The mean score for four Likert items ranged from 0.22 to 1.27 (SD ranged from 0.53 to 1.10), the mean score for three Likert items ranged from 0.30 to 0.88 (SD ranged from 0.32 to 1.88), and the mean score for five Likert items ranged from 0.83 to 1.38 (SD ranged from 1.03 to 1.44). The skewness values were all greater than 0 (range 0.14 to 3.17; *SD* = 0.077), and the kurtosis values ranged from −1.17 to 11.07 (*SD* = 0.153); for example, Item 38 had the highest skewness (3.17) and kurtosis (11.07).

**FIGURE 1:**
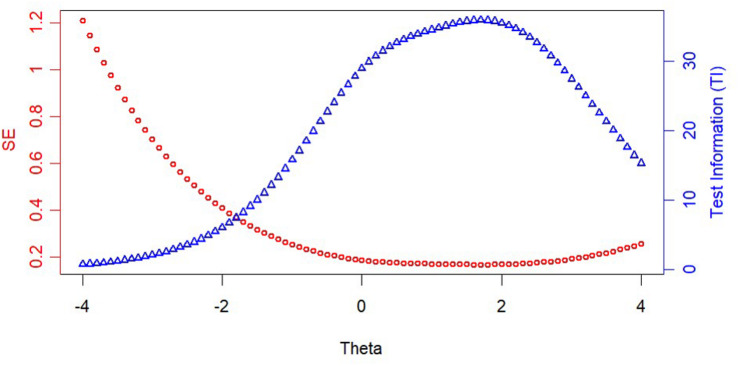
Test information and standard error (SE) curve of the CAT-SAD.

Figure 1 displays the reliability and test information of the final CAT-SAD item bank for the final estimate under stopping rule SE (θ) < 0.3. Furthermore, the precision of test information function was expounded, which measured adolescents’ latent traits whose location given was estimated as well. Figure 1 shows that the CAT-SAD provided ideal test information quantity on the latent trait ranging from −2 to 4.

#### Validity

The Pearson correlations between the full-scale SAAS-C score and the estimated score under different stopping rules (*SE* < 0.3, *SE* < 0.4, and *SE* < 0.5) for the CAT-SAD were 0.705, 0.685, and 0.650, respectively. These high or moderate significant correlations indicated that the CAT-SAD had acceptable criterion-related validity with the SAAS-C. In addition, the final item bank with 68 items covered all symptoms of SAD, as defined in the *DSM-5*, and each symptom was assessed by at least seven items. Therefore, the CAT-SAD also had acceptable content validity.

#### Sensitivity and Specificity

**FIGURE 2:**
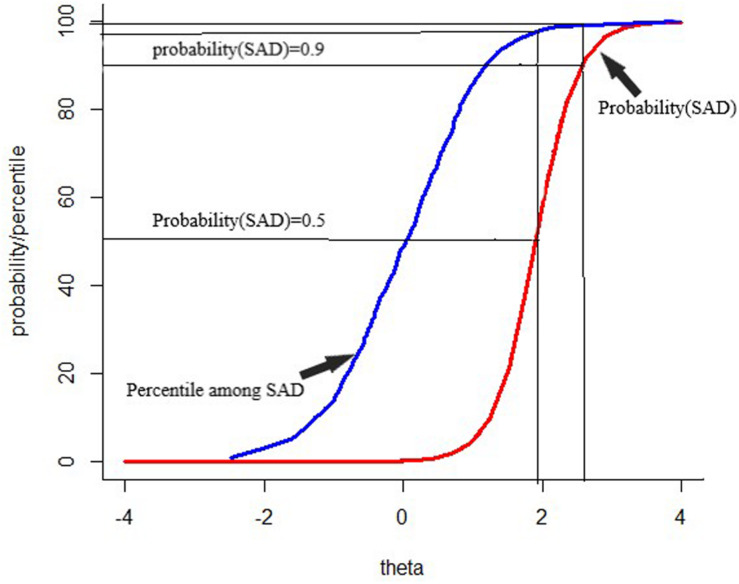
Percentile rank among patients with separation anxiety disorder and probability of separation anxiety disorder diagnosis for the range of scores on the computerized adaptive testing–separation anxiety disorder.

To make the scores more intuitive, the CAT-SAD scores, which used an average of 17 adaptively administered items [*SE* (θ) < 0.3], were strongly related to total SAAS-C scores (*r* = 0.706, *p* < 0.001). This relationship is shown in Figure 2. Figure 2 also displays the CAT-SAD score percentile ranking for adolescents who were classified as having SAD by the SAAS-C. For example, an adolescent with a CAT-SAD score of 1.78 had a 0.50 probability of meeting the SAD criteria—specifically, at the upper 94th percentile of the CAT-SAD distribution. In contrast, if an adolescent had a CAT-SAD score of −0.32, the probability of meeting criteria for SAD was Close to 0, and would be at the 50th percentile for the sample of adolescent.

The results of the sensitivity and specificity for CAT-SAD under different stopping rules are displayed in Table 5. The CAT-SAD area under the curve (AUC) value, based on the SAAS-C scale, was 0.958 under the “none” stopping rule (sensitivity = 0.900, specificity = 0.925, YI = 0.825), 0.925 under the stopping rule SE (θ) < 0.3 (sensitivity = 0.850, specificity = 0.900, YI = 0.749), 0.921 under the stopping rule SE (θ) < 0.4 (sensitivity = 0.850, specificity = 0.865, YI = 0.714), and 0.912 under the stopping rule SE (θ) < 0.5 (sensitivity = 0.900, specificity = 0.815, YI = 0.715). Overall, the sensitivity and specificity under different stopping rules were acceptable. Taking CAT-SAD under the stopping rule of SE (θ) < 0.3 as an example, the SAAS-C scale was regarded as the classification criteria of SAD in which sensitivity was 0.850 and specificity was 0.900.

## Discussion

In this study, the steps to establish an item bank in a Chinese sample were unidimensionality, IRT model selection, item fit, DIF, and discrimination; the development of the CAT-SAD used a GRM to conduct simulation research. To obtain high-quality CAT-SAD development, the item bank consisted of six subscales to measure SAD, which comprehensively covered all criteria for adolescents with SAD per the *DSM-5*. Then, the most appropriate model could be selected from four common IRT models based on real data when strict unidimensionality was met. Results revealed that the final item bank included 68 items, the ratio between the first eigenvalue and the second eigenvalue displayed strict unidimensionality, and each symptom (which had eight criteria of separation anxiety per the *DSM-5*) was assessed by at least seven items. Further, the *S-X^2^* of the 68 items fit the GRM well, and the IRT discrimination of the item bank exhibited that the final item bank of the CAT-SAD was high quality.

Although the item bank contains eight symptoms of SAD, which all measure the same latent factor (i.e., SAD), the EFA demonstrated that the item bank formed six scales, and thus, SAD was unidimensional. Consistently, the first and second eigenvalues and first factor variance that was accounted for conformed to the standards of unidimensionality (Reckase, 1979).

The length of measurement can vary during the CAT process; therefore, the number of items and items answered by each participant differed. Further investigations presented that (1) the CAT-SAD had an acceptable marginal reliability, (2) the CAT-SAD had reasonable and acceptable criterion-related validity with the SAAS-C, (3) the sensitivity and specificity of the CAT-SAD were both acceptable under stopping rule *SE* < 0.3, and (4) the ROC curves showed that the AUC had an appropriate range under different stopping rules. Further, the number of items managed under the CAT format has been reduced by an average of 75% compared with P&P tests, and the correlation between scores obtained from the CAT-SAD and P&P tests was high and significant, which indicates that there is no significant loss of information. Consequently, the CAT-SAD is an effective and efficient measure to screen for varying degrees of SAD among adolescents, even without clinician assistance.

The scientific contribution of this study lies in the fact that we discovered an efficient method to assess SAD among adolescents that reduces the time and number of items to complete as compared to earlier measures. The test results have certain reference values for patients when they visit doctors; e.g., patients with mood disorders, who are difficult to assess over the long-term, can benefit from the efficiency of the CAT-SAD. Additionally, studies have shown that the suspension rule of *SE* < 0.3 is feasible for using CAT with adolescents, which has high validity, sensitivity, and specificity.

Of course, some limitations of this study are worth mentioning. First, concerning participant distribution, the number of abnormal participants obtained was very small, and the sample coverage was not diverse enough. In future studies, the sample distribution should be expanded to improve the representation of adolescents in cross-cultural studies of separation anxiety. Second, the title of the test bank targeted all participants, which may generate systematic bias when using CAT. Third, this research only notes CAT simulations; in the future, researchers should thoroughly validate the efficiency of the CAT-SAD in large-scale clinical trials; the simulated and actual CAT administration may have different results because there are many factors, such as answer time, individual emotion, test environment, and so on, that can affect individual responses in actual situations (Smits et al., 2011). Fortunately, as Kocalevent et al. (2009) showed that the simulated CAT and the actual CAT results were consistent, this paper still has some practical significance. However, the item bank can be used to construct short forms in situations in which researchers lack the equipment to complete a CAT, that is, to select a fixed set of items for optimal measurement in future studies. Indeed, CAT is supported for use in a special group (SAD) to investigate its practicality. Lastly, although the results showed that a test database established with the one-dimensional CAT can effectively diagnose SAD among adolescents, we focused only on diagnostic classification, which is of great help in clinical treatments, but the cognitive process mechanism underlying SAD remains unclear. In the future, the researchers, through cognitive diagnosis, can explore the cognitive process mechanism of SAD. SAD’s attributes are multidimensional, and it is difficult to determine which attributes have caused the patient to suffer from SAD. The CAT-SAD provides certain item bank information for the cognitive diagnosis of SAD, which can diagnose attributes for each patient quickly and also improve the efficiency and help the treatment.

SAD is one of the most common mental disorders among children and adolescents, and it may seriously affect their growth, daily life, and learning. There are two ways to diagnose SAD: clinical diagnosis based on doctors’ experience-based assessment and measurement. Nevertheless, the feasibility of clinical diagnosis has been questionable in some psychiatric and mental health clinics. Thus, it was necessary to relieve the pressure through measurement based on experience assessment. Psychometric tools are effective ways to screen for mental disorders in the field of clinical and mental health. This article reported on the development of a CAT version of SAD that involves shorter and more effective tools to measure SAD and analyze teenagers’ characteristics. Self-report scales, which require considerable time and personnel, have previously been used for diagnosis. The CAT-SAD could be used as a routine clinical assessment, to save clinicians’ time and ease patients’ burden. At the same time, it can serve as a tool for follow-up treatment and effective review. Moreover, the CAT-SAD can measure SAD for all Chinese adolescents, regardless of region, gender, age, or health condition. The current research provides an efficient and accurate psychometric tool for researchers and clinicians to measure SAD among adolescents. At present, there is no research, other than this paper, on the CAT version of SAD with Chinese adolescents. Of course, this study used well-known international SAD; therefore, the CAT-SAD may have some applicability to other countries’ adolescents.

## Data Availability Statement

The datasets generated in this article are not publicly available to maintain respondents’ anonymity. Requests to access the datasets should be directed to 651804834@qq.com.

## Ethics Statement

The studies involving human participants were reviewed and approved by the Research Center of Mental Health, Jiangxi Normal University. Written informed consent to participate in this study was provided by the participants’ legal guardian/next of kin.

## Author Contributions

YH: thesis writing. YC and DT: guided the thesis writing and data processing. YG and SL: data processing.

## Conflict of Interest

The authors declare that the research was conducted in the absence of any commercial or financial relationships that could be construed as a potential conflict of interest.

## References

[B1] Akaike H. T. (1974). A new look at the statistical model identification. *IEEE Trans. Automat. Contr.* 19 716–723. 10.1109/TAC.1974.1100705

[B2] Allen J. L. Lavallee K. L. Herren C. Ruhe K. Schneider S. (2010). DSM IV criteria for childhood separation anxiety disorder: informant, age, and sex differences. *J. Anxiety Disord.* 24 946–952. 10.1016/j.janxdis.2010.06.022 20675099

[B4] Baker F. B. (1992). *Item Response Theory: Parameter Estimation Techniques.* New York, NY: Marcel Dekker.

[B5] Battaglia M. Pesenti-Gritti P. Medland S. E. Ogliari A. Tambs K. Spatola C. A. M. (2009). A genetically informed study of the association between childhood separation anxiety, sensitivity to CO_2_, panic disorder, and the effect of childhood parental loss. *Arch. Gen. Psychiatry* 66, 64–71. 10.1001/archgenpsychiatry.2008.513 19124689

[B6] Birmaher B. Brent D. A. Chiappetta L. Bridge J. Monga S. Baugher M. (1999). Psychometric properties of the screen for child anxiety related emotional disorders (scared): a replication study. *J. Am. Acad. Child Adolesc. Psychiatry* 38 1230–1236. 10.1097/00004583-199910000-00011 10517055

[B8] Bock R. D. (1972). Estimating item parameters and latent ability when responses are scored in two or more nominal categories. *Psychometrika* 37 29–51. 10.1007/BF02291411

[B9] Bock R. D. Mislevy R. J. (1982). Adaptive eap estimation of ability in a microcomputer environment. *Appl. Psychol. Meas.* 6 431–444. 10.1177/014662168200600405

[B10] Bowlby J. (1960). Separation anxiety. *Int. J. Psychoanal.* 41, 89–113. 10.1080/00140136008930499 13803480

[B11] Brand S. Wilhelm F. H. Kossowsky J. Holsboer-Trachsler E. Schneider S. (2011). Children suffering from separation anxiety disorder (SAD) show increased HPA axis activity compared to healthy controls. *J. Psychiatr. Res.* 45 452–459. 10.1016/j.jpsychires.2010.08.014 20870248

[B12] Chalmers R. P. (2012). Mirt: a multidimensional item response theory package for the r environment. *J. Stat. Softw.* 48 1–29. 10.18637/jss.v048.i06

[B13] Chessa D. Di R. D. Delvecchio E. Lis A. (2012). Assessing separation anxiety in italian youth: preliminary psychometric properties of the separation anxiety assessment scale. *Percept. Mot. Skills* 115 811–832. 10.2466/03.10.15.PMS.115.6.811-832 23409595

[B14] Choi S. W. (2015). *Lordif: Logistic Ordinal Regression Differential Item Functioning Using IRT.Version 0.3-3.*

[B15] Chorpita B. F. Yim L. Moffitt C. Umemoto L. A. Francis S. E. (2000). Assessment of symptoms of DSM-IV anxiety and depression in children: A revised child anxiety and depression scale. *Behav. Res. Ther.* 38, 835–855. 10.1016/S0005-7967(99)00130-8 10937431

[B16] Costello E. J. Mustillo S. Erkanli A. Keeler G. Angold A. (2003). Prevalence and development of psychiatric disorders in childhood and adolescence. *Arch. Gen. Psychiatry* 60:837. 10.1001/archpsyc.60.8.837 12912767

[B17] Crane P. K. Gibbons L. E. Jolley L. Van Belle G. (2006). Differential item functioning analysis with ordinal logistic regression techniques: DIFdetect and difwithpar. *Med. Care* 44, S115–S123. 10.1097/01.mlr.0000245183.28384.ed 17060818

[B18] Eisen A. R. Schaefer C. E. (2007). Separation anxiety in children and adolescents: an individualized approach to assessment and treatment. *J. Can. Acad. Child Adolesc. Psychiatry* 16 39–41. 10.1089/cap.1999.9.277 2375736

[B19] Essau C. A. Sasagawa S. Anastassiou-Hadjicharalambous X. Guzmán B. O. Ollendick T. H. (2011). Psychometric properties of the spence child anxiety scale with adolescents from five european countries. *J. Anxiety Disord.* 25 19–27. 10.1016/j.janxdis.2010.07.001 20685072

[B20] Flens G. Smits N. Terwee C. B. Dekker J. Huijbrechts I. de Beurs E. (2017). Development of a computer adaptive test for depression based on the dutch-flemish version of the PROMIS Item Bank. *Eval. Health Prof.* 40 79–105. 10.1177/0163278716684168 28705028

[B21] Green B. F. Bock R. D. Humphreys L. G. Linn R. L. Reckase M. D. (1984). Technical guidelines for assessing computerized adaptive tests. *J. Educ. Meas.* 21, 347–360. 10.1111/j.1745-3984.1984.tb01039.x

[B22] Hahn I. Hajinlian J. Eisen A. R. Winder B. Pincus D. B. (2003). “Measuring the dimensions of separation anxiety and early panic in children and adolescents: the separation anxiety assessment Scale,” in *Paper Presented at the 37th Annual Convention of the Association for the Advancement of Behavior Therapy: Recent Advances in the Treatment of Separation Anxiety and Panic in Children and Adolescents*, ed. Eisen A. R., Boston, MA.

[B23] Hattie J. (1984). An empirical study of various indices for determining unidimensionality. *Multivariate Behav. Res.* 19, 49–78 10.1207/s15327906mbr1901_3 26776067

[B24] In-Albon T. Meyer A. H. Schneider S. (2013). Separation anxiety avoidance inventory-child and parent version: psychometric properties and clinical utility in a clinical and school sample. *Child Psychiatry Hum. Dev.* 44 689–697. 10.1007/s10578-013-0364-z 23385519

[B25] Jamshidian M. Jalal S. Jansen C. (2014). Missmech: an R package for testing homoscedasticity, multivariate normality, and missing completely at random (MCAR). *J. Stat. Softw.* 56, 1–31. 10.18637/jss.v056.i06

[B26] Kessler R. C. Chiu W. T. Demler O. Merikangas K. R. Walters E. E. (2005). Prevalence, severity, and comorbidity of 12-month DSM-IV disorders in the National Comorbidity Survey Replication (NCS-R). *Arch. Gen. Psychiatry* 62, 617–627. 10.1001/archpsyc.62.6.617 15939839 PMC2847357

[B27] Kocalevent R.-D. Rose M. Becker J. Walter O. B. Fliege H. Bjorner J. B. (2009). An evaluation of patient-reported outcomes found computerized adaptive testing was efficient in assessing stress perception. *J. Clin. Epidemiol.* 62, 278–287. 10.1016/j.jclinepi.2008.03.003 18639439

[B28] Kossowsky J. Wilhelm F. H. Roth W. T. Schneider S. (2012). Separation anxiety disorder in children: disorder-specific responses to experimental separation from the mother. *J. Child Psychol. Psychiatry* 53 178–187. 10.1111/j.1469-7610.2011.02465.x 21923807

[B29] Kreitzberg C. B. Jones D. H. (1980). An empirical study of the broad range tailored test of verbal ability. *ETS Res. Rep. Ser.* 1980 1–232. 10.1002/j.2333-8504.1980.tb01195.x

[B30] Last C. G. Perrin S. Hersen M. Kazdin A. E. (1992). Dsm-iii-r anxiety disorders in children: sociodemographic and clinical characteristics. *J. Am. Acad. Child Adolesc. Psychiatry* 31 1070–1076. 10.1097/00004583-199211000-00012 1429407

[B31] Lipsitz J. D. Martin L. Y. Mannuzza S. Chapman T. F. Liebowitz M. R. Klein D. F. (1994). Childhood separation anxiety disorder in patients with adult anxiety. *Am. J. Psychiatry* 151, 927–929. 10.1176/ajp.151.6.927 8185008

[B32] Lord F. M. (1980). *Applications of Item Response Theory to Practical Testing Problems*. New Jersey: Lawrence Erlbaum.

[B33] Magis D. Barrada J. R. (2017). Computerized adaptive testing with R: recent updates of the package catR. *J. Stat. Softw.* 76, 1–19. 10.18637/jss.v076.c01

[B34] Magis D. Raiche G. (2012). Random generation of response patterns under computerized adaptive testing with the R package catR. *J. Stat. Softw.* 48, 1–31. 10.18637/jss.v048.i08

[B35] Manicavasaga V. Silove D. Curtis J. (1997). Separation anxiety in adulthood: a phenomenological investigation. *Compr. Psychiatry* 38, 274–282. 10.1016/s0010-440x(97)90060-2 9298320

[B36] Merikangas K. R. He J.-P. Brody D. Fisher P. W. Bourdon K. Koretz D. S. (2010). Prevalence and treatment of mental disorders among US children in the 2001–2004 NHANES. *Pediatrics* 125, 75–81. 10.1542/peds.2008-2598 20008426 PMC2938794

[B37] Milrod B. Markowitz J. C. Gerber A. J. Cyranowski J. Altemus M. Shapiro T. (2014). Childhood separation anxiety and the pathogenesis and treatment of adult anxiety. *Am. J. Psychiatry* 171, 34–43. 10.1176/appi.ajp.2013.13060781 24129927

[B38] Muraki E. (1992). A generalized partial credit model: application of an EM algorithm. *Appl. Psychol. Meas.* 16, 159–176. 10.1002/j.2333-8504.1992.tb01436.x

[B39] Nunnally J. C. (1978). Psychometric theory. *Am. Educ. Res. J.* 5:83 10.2307/1161962

[B40] Orlando M. Thissen D. (2000). Likelihood-based item-fit indices for dichotomous item response theory models. *Appl. Psychol. Meas.* 24 50–64. 10.1177/01466216000241003

[B41] Orlando M. Thissen D. (2003). Further investigation of the performance of S-X2: an item fit index for use with dichotomous item response theory models. *Appl. Psychol. Meas.* 27 289–298. 10.1177/0146621603027004004

[B42] Purperouakil D. Franc N. (2010). [Separation anxiety in children]. *La Revue Du Praticien* 60 783–787. 20623893

[B43] Reckase M. D. (1979). Unifactor latent trait models applied to multifactor tests: Results and implications. *J. Educ. Stat.* 4, 207–230. 10.3102/10769986004003207

[B44] Rubin D. B. (1976) Inference and missing data. *Biometrika* 63, 581–592. 10.1093/biomet/63.3.581

[B45] Samajima F. (1994). Estimation of reliability coefficients using the test information function and its modifications. *Appl. Psychol. Meas.* 18 229–244. 10.1177/014662169401800304

[B46] Samejima F. (1969). Estimation of latent ability using a response pattern of graded scores. *Psychometrika* 34, 1–97. 10.1007/BF03372160

[B47] Schisterman E. F. Perkins N. J. Liu A. Bondell H. (2005). Optimal cut-point and its corresponding Youden Index to discriminate individuals using pooled blood samples. *Epidemiology* 16 73–81. 10.1097/01.ede.0000147512.81966.ba 15613948

[B48] Schneider S. In-Albon T. (2005). *Separation Anxiety Avoidance Inventory, Child and Parent Version.* Basel: University of Basel. 10.1007/s10578-013-0364-z 23385519

[B49] Schwarz G. (1978). Estimating the dimension of a model. *Ann. Stat.* 6 461–464. 10.1214/aos/1176345415

[B57] Shear K. Jin R. Ruscio A. M. Walters E. E. Kessler R. C. (2006). Prevalence and correlates of estimated DSM-IV child and adult separation anxiety disorder in the national comorbidity survey replication. *Am. J. Psychiatry* 163, 1074–1083. 10.1176/ajp.2006.163.6.1074 16741209 PMC1924723

[B50] Silove D. Manicavasagar V. O’Connell D. Blaszczynski A. Wagner R. Henry J. (1993). The development of the separation anxiety symptom inventory (sasi). *Aust. N. Z. J. Psychiatry* 27 477–488. 10.3109/00048679309075806 8250793

[B51] Smits N. Cuijpers P. Straten A. V. (2011). Applying computerized adaptive testing to the CES-D scale: a simulation study. *Psychiatry Res.* 188 147–155. 10.1016/j.psychres.2010.12.001 21208660

[B52] Spence S. H. (1998). A measure of anxiety symptoms among children. *Behav. Res. Ther.* 36 545–566. 10.1016/S0005-7967(98)00034-5 9648330

[B53] Spence S. H. Barrett P. M. Turner C. M. (2003). Psychometric properties of the spence children’s anxiety scale with young adolescents. *J. Anxiety Disord.* 17 605–625. 10.1016/S0887-6185(02)00236-0 14624814

[B54] Tan Q. Cai Y. Li Q. Zhang Y. Tu D. (2018). Development and validation of an item bank for depression screening in the Chinese population using computer adaptive testing: a simulation study. *Front. Psychol.* 9:1225. 10.3389/fpsyg.2018.01225 30072935 PMC6058179

[B55] Zhao J. Xing X. Wang M. (2012). Psychometric properties of the spence children’s anxiety scale (scas) in mainland chinese children and adolescents. *J. Anxiety Disord.* 26 728–736. 10.1016/j.janxdis.2012.05.006 22858899

[B56] Zumbo B. D. (1999). *A Handbook on the Theory and Methods of Differential Item Functioning (DIF): Logistic Regression Modeling as a Unitary Framework for Binary and Likert-Type (Ordinal) Item Scores.* Ottawa, ON: National Defense Headquarters.

